# Enhancing seafood traceability: tracking the origin of seabass and seabream from the tuscan coast area by the analysis of the gill bacterial communities

**DOI:** 10.1186/s42523-024-00300-z

**Published:** 2024-03-14

**Authors:** Niccolò Meriggi, Alessandro Russo, Sonia Renzi, Benedetta Cerasuolo, Marta Nerini, Alberto Ugolini, Massimiliano Marvasi, Duccio Cavalieri

**Affiliations:** 1 Institute of Agricultural Biology and Biotechnology (IBBA), National Research Council (CNR), Pisa, IT56124 Italia; 2 Department of Biology, University of Florence, Sesto Fiorentino, IT50019 Italy; 3 Department of Biology, University of Florence, Florence, IT50125 Italia

## Abstract

**Background:**

The seafood consumption and trade have increased over the years, and along its expected expansion pose major challenges to the seafood industry and government institutions. In particular, the global trade in fish products and the consequent consumption are linked to reliable authentication, necessary to guarantee lawful trade and healthy consumption. Alterations or errors in this process can lead to commercial fraud and/or health threats. Consequently, the development of new investigative tools became crucial in ensuring unwanted scenarios. Here we used NGS techniques through targeted metagenomics approach on the V3-V4 region of the 16S rRNA genes to characterize the gill bacterial communities in wild-caught seabream (*Sparus aurata*) and seabass (*Dicentrarchus labrax*) within different fisheries areas of the “Costa degli Etruschi’’ area in the Tuscan coast. Our challenge involved the possibility of discriminating between the microbiota of both fish species collected from three different fishing sites very close to each other (all within 100 km) in important areas from a commercial and tourist point of view.

**Results:**

Our results showed a significant difference in the assembly of gill bacterial communities in terms of diversity (alpha and beta diversity) of both seabass and seabream in accordance with the three fishing areas. These differences were represented by a unique site -related bacterial signature, more evident in seabream compared to the seabass. Accordingly, the core membership of seabream specimens within the three different sites was minimal compared to the seabass which showed a greater number of sequence variants shared among the different fishing sites. Therefore, the LRT analysis highlighted the possibility of obtaining specific fish bacterial signatures associated with each site; it is noteworthy that specific taxa showed a unique association with the fishing site regardless of the fish species. This study demonstrates the effectiveness of target-metagenomic sequencing of gills in discriminating bacterial signatures of specimens collected from fishing areas located at a limited distance to each other.

**Conclusions:**

This study provides new information relating the structure of the gill microbiota of seabass and seabream in a fishing area with a crucial commercial and tourist interest, namely “Costa degli Etruschi”. This study demonstrated that microbiome-based approaches can represent an important tool for validating the seafood origins with a central applicative perspective in the seafood traceability system.

**Supplementary Information:**

The online version contains supplementary material available at 10.1186/s42523-024-00300-z.

## Background

Globalization has enabled access to a wide variety of food resources around the world, however it has imposed new challenges for the control of food supply chains and food security. These conditions are of greatest interest when we deal with seafood, whose consumption covers nearly one fifth of world animal protein intake. Indeed, the consumption rate of aquatic foods worldwide has doubled in the last 50 years [1], highlighting the impact on the economy and public health that a possible problem in the trade chain could cause. The perishability of seafood requires a higher accuracy in food safety control procedures and its importance in the global food trade impose the development of new methodologies to ensure errors or fraud in the global supply chain [2]. Traceability plays a crucial role in supporting the sustainability of fisheries by providing transparency, accountability, and the ability to monitor and manage fishing activities effectively [3]. Traceability supports sustainability in fisheries in many ways: i) it helps to prevent Illegal, Unreported, and Unregulated (IUU) fishery which depletes natural fish stocks (EC No 1005/2008 of 29 September 2008); (ii) improve consumer confidence and market access, including production such as wild-caught or farmed [4]; (iii) supports certifications programmes that demonstrate commitment to sustainable practices and gain access to niche markets that prioritize environmentally responsible products (EU No 664/2014 of 18 December 2013), (iv) traceability allows for better management of the supply chain, reducing the likelihood of fish spoilage and waste [5]. Finally, traceability technologies will provide valuable information for managers and policymakers to assess the health of seafood stocks and determine sustainable catch levels [6]. The criteria for the correct tracking of seafood products are regulated by Council Regulation (EC) No. 1379/2013 and regard the common procedures of the markets in fishery and aquaculture products. The great attention exerted by the control authorities is not determined only by the risk of seafood contamination or wrong preservation but is also aimed at guaranteeing the rights of consumers and producers who aim to request certifications of origin.

Consequently, one of the main challenges in the seafood industry regards the possibility of tracking the product’s origin. The regulatory infrastructure is not sufficient to guarantee traceability without suitable technological and analytical support. In this direction, the development and adoption of new methodological approaches for the identification of fishes from different areas can represent a central resource in traceability management.

Microbial communities analysis of individuals from different habitats demonstrated the forensic value of this type of approach to obtain distinctive signatures representative of specific environments. Actually, an increasingly consistent number of different approaches were explored to trace seafood origins [10, 11], these approaches include DNA metabarcoding approaches, stable isotope analysis or machine learning approaches based on sequencing data [2, 12–14]. Next Generation Sequencing (NGS) techniques represent an extremely interesting tool as they are able to provide a comprehensive characterization of the microbial communities of the area of origin. The progressive reduction of the cost of these methodologies has made the NGS approaches a tool that can be easily used by regulatory authorities, control organisms and companies.

If we consider the approaches based on the analysis of bacterial DNA sequences, a wide number of these usually include PCR-DGGE-based analysis [15], however the NGS approaches along with the possibility to obtain high-throughput and more accurate information regarding microbial signature [16] has progressively replaced the PCR-DGGE, which it was commonly used in a wide number of case studies [16, 17]. NGS provides, conversely to PCR-DGGE, higher resolution leading to the microbial species identifications [18], detection of a wide range of microorganisms or genetic variations [19], last but not least, the data generated by NGS can be easily stored for future reference and used for comparisons against potential fraudulent products. According to this evidence, the exploitation of microbial metagenomics tools for traceability has been proposed to assess the origin of food in other scenarios [16, 20].

Targeted-metagenomic approaches can also offer solutions in ensuring health safety and sustainable consumption. In fact, these techniques allow to follow the variations in the microbial communities as indicators of deterioration, defined as specific spoilage-associated organisms (SSOs) [7, 8]. This allows to improve the management of fish resources, facilitating the control of deterioration processes within the fish supply chain. The possibility of determining SSOs, combined with the possibility of discriminating the origin of the fish both at a local and higher spatial levels [9] can be useful to guarantee not only the origin but also safety and sustainable seafood consumption. In fact, although European legislation requires the affixing of labels to certifying the area of origin according to the The Food and Agriculture Organization (FAO) tracking system (see materials and methods section for details on FAO references), it is difficult to estimate the freshness of the fish catch which, in fact, depends on conservation parameters not necessarily related to the local or foreign origin of fish caught [9]. Thus, determining the microbial composition associated with a given specimen, with possible SSOs identification, can increase the food safety, providing guarantees of sustainable consumption, allowing the consumer to be aware of the origin and health status of the fish product, even at a local level.

The scientific literature is mainly represented by studies on the fish intestinal microbiota [21], although the gills represent an ideal tissue for studying fish traceability given the high degree of variability of the resident microbial communities following environmental variations [22]. Few studies highlight the central role of gills in assessing the environmental microbial signature (useful for traceability): The gill microbiota, then, could be a representative metric for interpreting the environmental microbial signature as highlighted in a recent study on seabream which showed that gill microbiota is more closely reflecting the environment than other organs (gut, skin, fillet) [23]. Therefore, the gills are receiving increasing attention, as they may be sampled without destroying or compromising the saleable parts of the fish (i.e. by swabbing or biopsy) [24, 25]. In this context, we found several studies focused on the microbiota of molluscs [14, 26], while those that analyzed fish concerned farmed specimens [16]. We believe that the challenge of these techniques represents the understanding of their applicability on on-board frozen fish caught in open-sea areas, recreating a step of control of the area of origin within the fish supply chain.

Here, we analyzed the gill microbiota of two fish species of central interest in the global fish trade, i.e. *Dicentrarchus labrax* (hereafter seabass) and *Sparus aurata* (hereafter seabream), caught in the Mediterranean Sea from three different areas within the area of Tuscan coast named “Costa degli Etruschi”. We performed 16 S rRNA gene sequencing (V3-V4 region) by using targeted-metagenomic approach. We describe for the first time the gill microbial profile of the seabass and seabream specimens from this particular fishing area, investigating interspecific and intraspecific bacterial variations of wild specimens caught at a very limited geographical distance. Our data suggest the possibility to discriminate between wild fish species according to their sampling area despite limited geographical resolution.

## Results

### Different fishing site determine different gills bacterial diversity assemblage

**Fig. 1:**
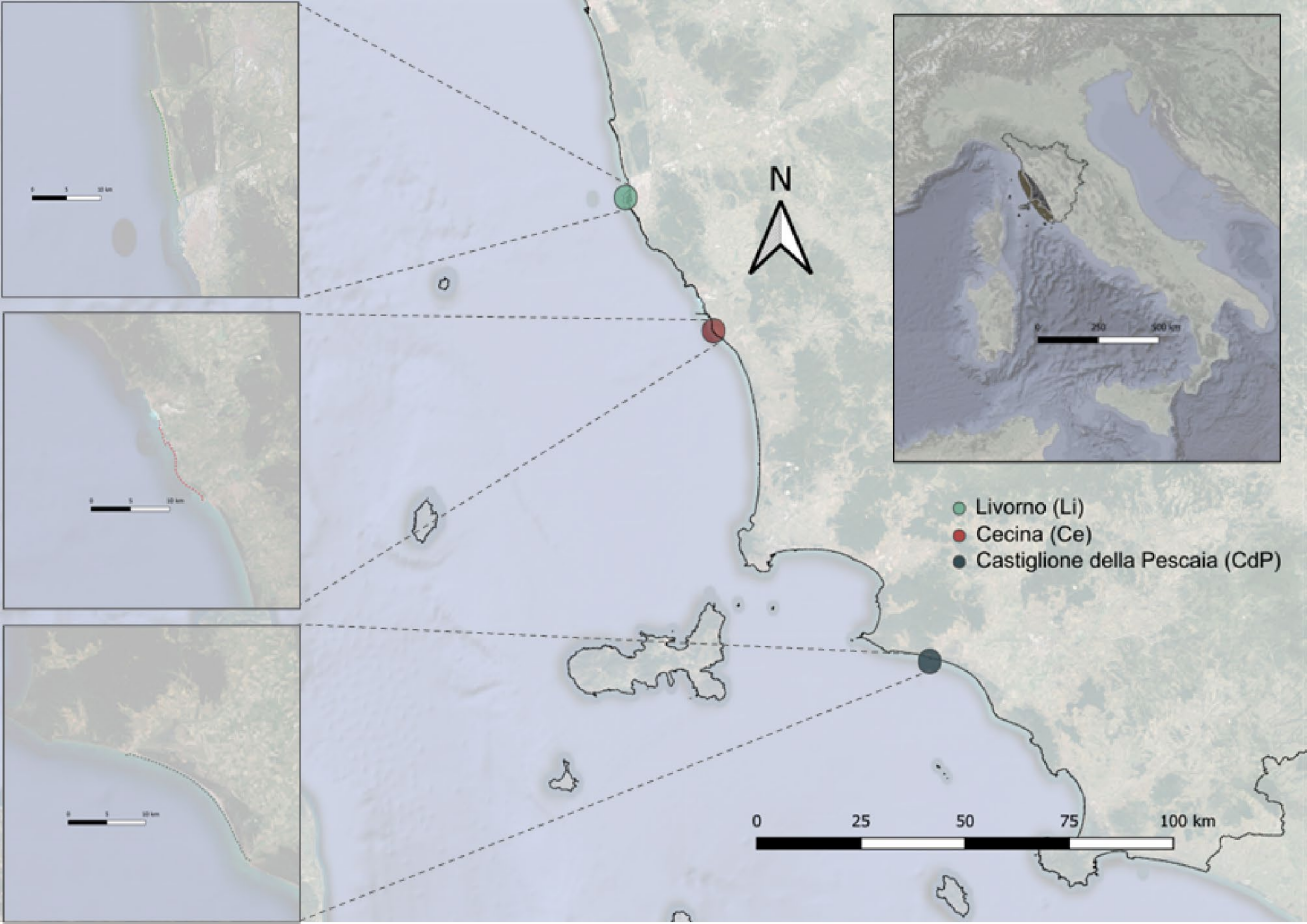
Geographical distribution of fishing areas in which the populations of the two fish species *D. labrax* and *S. aurata* are caught. The map shows the sampling sites (color code) according to the name of the related fishing port. The enlargements of the parts of the coast are shown on the left of the map and refer to the coordinates reported in the materials and methods section. Map of Tuscan coast is generated using QGIS version 3.30 (https://qgis.org) and edited using open source graphics editor Inkscape 1.1.2 (https://inkscape.org/) highlighting the specific fishing areas according to the legend mentioned in the figure

After DADA2 analysis pipeline and subsequent quality filtering a total of 4’162’462 reads (median: 72’787.5) clustered in 8’736 different bacterial amplicon sequence variants (ASVs) were detected. Seabass and seabream datasets were characterized by a significantly different number of ASVs and related extrapolated diversity (Inverse Simpson index) highlighting significant differences in the structure of the gill microbiota between the two fish species (Fig. S1c-d). To exclude a methodological bias the rarefaction curves and Good’s coverage estimation were calculated. Rarefaction curves reached the plateau condition demonstrating the effectiveness of sequencing in describing the bacterial diversity of each sample (Fig. S1a) and this was corroborated by the Good’s coverage estimator which ranged from 99.99 to 100% (Fig. S1b). All the following analyses were carried out in order to highlight variations in the gills bacterial structure of the two fish species, between the three different coastal sites within the area “Costa degli Etruschi” in accordance with the experimental design in Fig. 1.

**Fig. 2:**
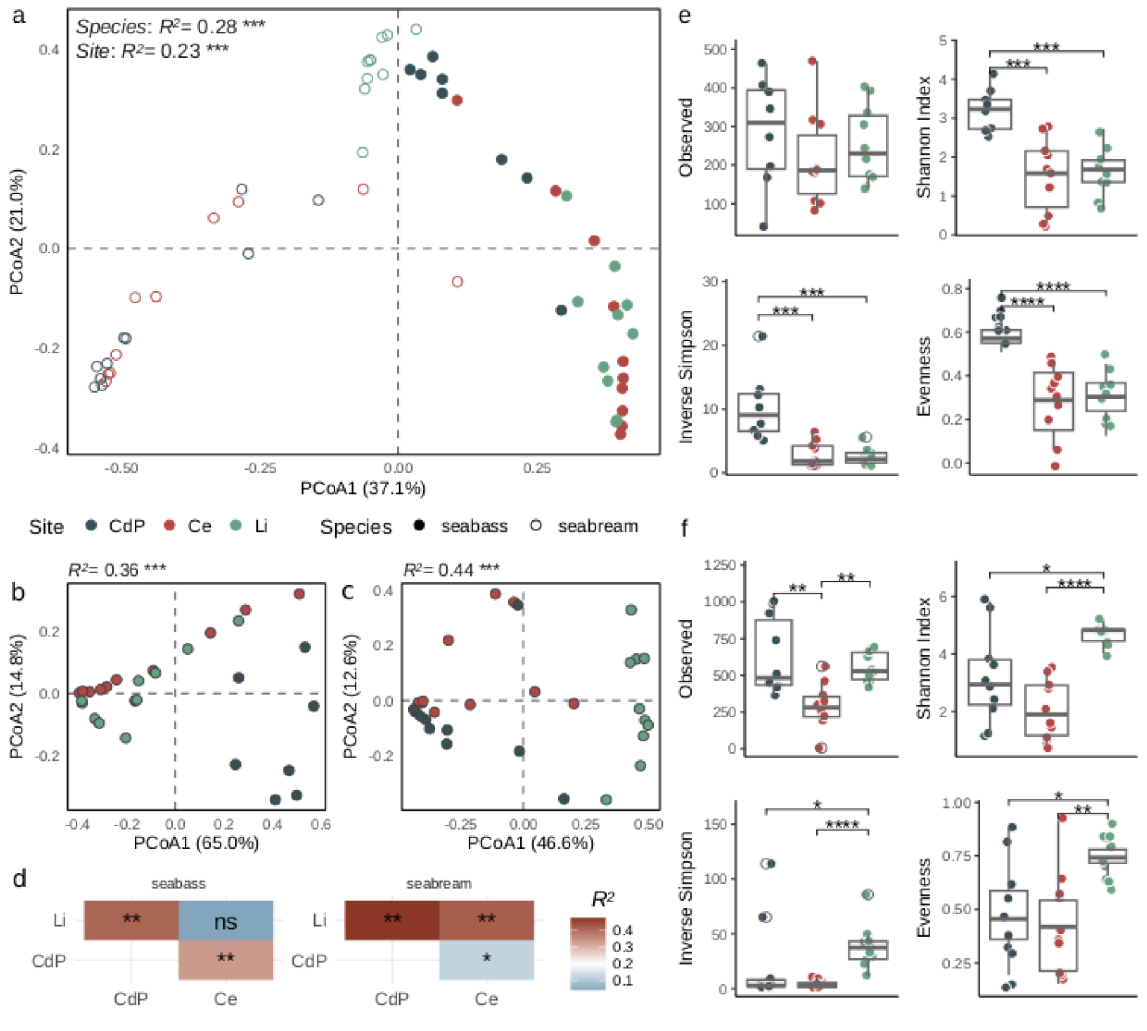
Bacterial distribution among sites in seabass and seabream. **a** Multidimensional scaling analysis (PCoA) based on Bray-Curtis distance according to the main two variables, fish species (shape pattern) and site (color pattern). R-squared values and significance after adonis permanova analysis tested on *species* and *site* variables are reported inside the panel. **b**, **c** PCoA based on Bray-Curtis distance performed after splitting the dataset according to the fish species, i.e. seabream (**b**) and seabass (**c**). PCoA reported sample distribution according to the different sites (color pattern) while R-squared values and significance from adonis permanova analysis are reported on top of the panels. **d** Heatmaps display statistical significance and R-squared values from pairwise adonis permanova for each comparison between groups, from lower value (blue scale) to higher value (red scale). Significant pairs are reported using asterisks (ns; not significative, *; *P* < 0.05, **; *P* < 0.01). **e**, **f** Boxplots reported the alpha diversity metrics among different sites in seabass (**e**) and seabream (**f**) datasets. Pairwise comparisons among alpha diversity measures were calculated by using the Wilcoxon test (Benjamini-Hochberg adjustment). Significant pairs are reported using asterisks (*; *P* < 0.05, **; *P* < 0.01, ***; *P* < 0.001, ****; *P* < 0.0001). The fishing sites are reported according to the following legend, CdP: Castiglione della Pescaia, Ce: Cecina, Li: Livorno

The multidimensional analysis (PCoA based on Bray-Curtis distance) showed a sharp separation between samples according to both fish species and collection sites variables (Fig. 2a). The fish species represents the main variable in the assembly of gills bacterial diversity in accordance with the explained variance results from adonis permanova (*R*^*2*^ in Fig. 2a). The fish species and fishing site variables are effective in describing around the 28% and 23% of the variance explained, respectively (*R*^*2*^ in Fig. 2a). To clearly understand the actual diversity between different collection sites, we divided the dataset in accordance to the two different fish species, then we calculated diversity metrics and performed multivariate analysis as described above.

Multivariate analysis (PCoA based on Bray-Curtis distance) produced a clear separation among groups for almost all fishing sites in the two fish species datasets (Fig. 21b-c). The overall effect of site variable, assessed by adonis permanova, suggested a higher impact of different fishing sites in driving gills diversity of seabream compared to seabass (*R*^*2*^ in Fig. 2b-c). To test the significant differences between the individual sites, the pairwise adonis permanova analysis was performed for both seabass and seabream datasets. The analysis showed significant differences in diversity between samples from different fishing sites for both fish species datasets, with the exception of the comparison between samples collected in Ce site compared to Li site in seabass dataset (Fig. 2d and Table S2).

Differences in alpha diversity metrics among different fishing sites for both fish species were also estimated. Bacterial diversity (alpha diversity) was rather uniform in seabass caught in Ce site and Li site while whole diversity measures reported significantly higher values in CdP site (Fig. 2e). No significant differences in the total number of bacterial ASVs were observed in seabass caught from the three different sites (Observed in Fig. 2e). The alpha diversity metrics in seabream dataset showed a more heterogeneous pattern, reporting a significantly lower number of bacterial ASVs in fish collected in Ce site compared to the others, whereas fish collected in Li site reported significantly higher diversity measure rates compared to the others (Fig. 2e). Therefore, the diversity metrics (beta and alpha) represented a functional discrimination criterion to map the gills bacterial signatures of different fish species from different fishing sites.

### The gill bacterial signature switch according to different fishing site

**Fig. 3:**
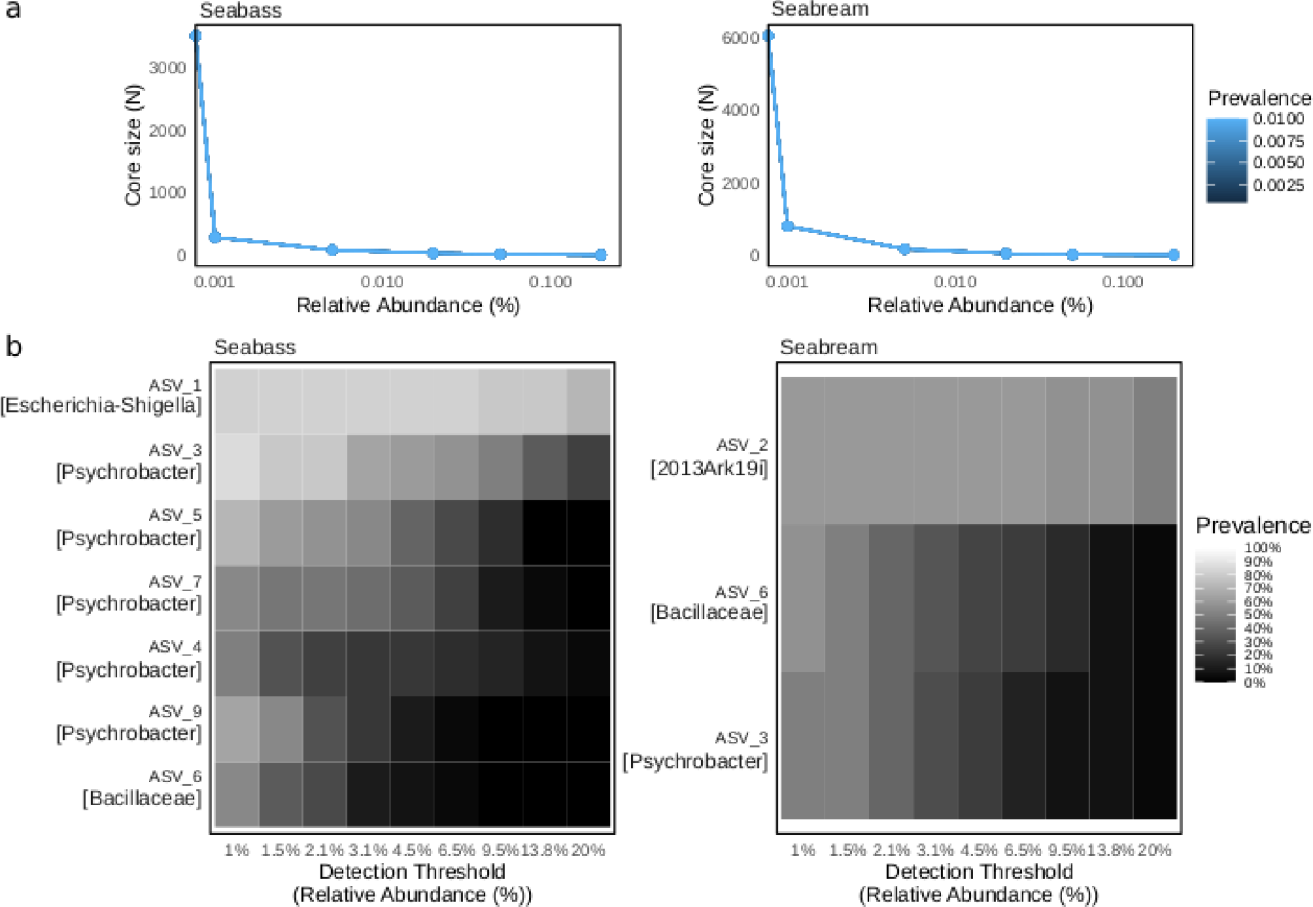
Bacterial ASV-level core microbiota. **a** Line chart shows the trend of core ASVs with their prevalence (color gradient) across the relative abundance thresholds. **b** Heatmap reports the core ASVs and related taxonomic assignment with their prevalence (color gradient) at different detection thresholds (Relative abundance) in each fish species dataset. The y-axis reports the prevalence rate of the core ASVs while the x-axis reports the different detection thresholds (relative abundance) range

In order to inspect the core membership in the two fish species we performed an ASV-level core microbiome analysis regardless of the fishing site (Fig. 3). The assembly of gills bacterial communities was strongly dependent on the different fishing sites and was highlighted by the overall limited number of core members in both fish species datasets (Fig. 3a).

The ASV_1 (*Escherichia-Shigella*) represented the widely shared variant in the core gills microbiota of seabass present in all fishing sites while the other core variants were largely associated with the *Psychrobacter* genus (Seabass in Fig. 3b). The seabream showed a higher variability in the gills microbiota according to each fishing sites and this was evidenced by the reduced core microbiota, composed by three variants only, among these the most widely represented was the ASV_2 (*2013Ark19i*) (Seabream in Fig. 3b).

We also estimate occupancy-abundance metrics for each sample site in both fish species to better describe the fish species core membership. The analysis did not explore the direct contribution to beta diversity but characterized the most representative ASVs for each site within each fish species considering their occupancy-abundance ratio (Fig. S2 and Additional file 1: Table S3). To deepen the taxonomic variability associated with each site we highlighted the ASVs with occupancy = 1, i.e. present in 100% of the samples associated with the site (The top 10 ASVs with relative taxonomic assignment were represented in Fig. S3). The analysis showed that some taxa are representative of the gills core membership of the fish species thus detectable in all sites within the fish species dataset, this condition was evident for the genus *Psychrobacter* (Additional file 1: Table S3). However, we observed that specific ASVs represent the core features (Occupancy = 1) of one specific site only within specific fish species dataset. Considering only some random examples, ASV_85 (*Halobacillus*), ASV_114 (*Cyanobium PCC- 6307*), ASV_283 (*Aurantimonas*) and ASV_299 (*Fictibacillus*) represented the core members of CdP site in seabream dataset only, while ASV_33 (*Carnobacterium*) and ASV_38 (*Lysinibacillus*) represented the core members of Ce in seabass dataset only.

These results corroborate the presence of a taxonomic site-related variability in both seabass and seabream datasets thus, highlighting the presence of specific core membership indicative of different fishing sites.

**Fig. 4:**
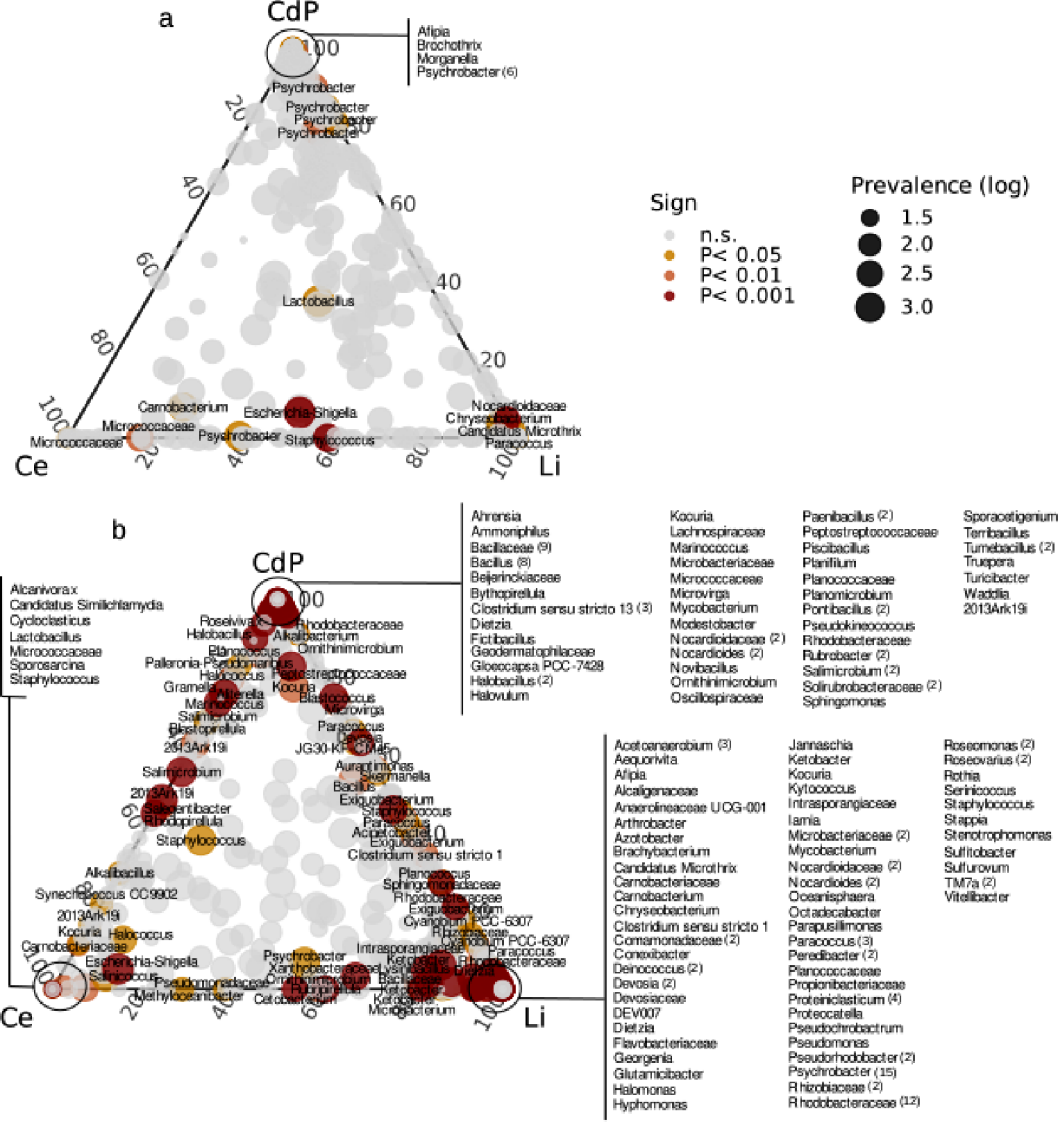
bacterial profiles switch according to different fishing sites. Ternary plots report the ASVs mean relative abundances distribution with the logarithm of prevalence (size scale). The color scheme represents ASVs significantly associated with different fishing sites according to LRT analysis in seabass (**a**) and seabream (**b**) datasets. For each significant ASVs the deepest taxonomic assignment is also reported. The brackets together with the taxonomic description highlight the number of ASVs detected for the same taxonomic assignment. The fishing sites are reported according to the following abbreviation, CdP: Castiglione della Pescaia, Ce: Cecina, Li: Livorno

In order to inspect bacterial ASVs distribution in different sites, the log-likelihood ratio test (LRT of DESeq2) on both fish species dataset was performed (Additional file 2: Table S4). We detect a total of 24 and 247 different ASVs for seabass and seabream datasets, respectively (Additional file 2: Table S4). The ASVs mean abundance were calculated together with ASVs prevalence (see materials and methods for details) and represented within ternary space according to the three fishing sites (Fig. 2a-b), highlighting the ASVs significantly selected by LRT analysis (Fig. 4). The distribution of ASVs significantly influenced by fishing sites (see colored scheme in Fig. 4 and clustering analysis in Fig. S4 and Additional file 3: Fig. S5) based on their abundance showed a taxonomic variability consistent with different fishing sites.

This analysis suggested the presence of the association among fishing sites and specific taxonomic features. Considering the seabass dataset, a total of 10 different ASVs assigned to the *Psychrobacter* genus were significantly associated with the CdP site while the *Carnobacterium* and *Micrococcaceae* phylotypes were associated with the Ce site whereas *Chryseobacterium*, *Candidatus Microthrix* and *Paracoccus* genera with the Li site (Fig. 4a). The same condition was depicted in the seabream dataset, with the presence of a large number of genera exclusively associated with a fishing site, among these some random examples include, the genera *Ahrensia* and *Alkalibacterium* significantly associated with the CdP site, the genera *Alcanivorax* and *Cycloclasticus* significantly associated with the Ce site and the *Cnexibacter* and *Glutamicibacter* genera were significantly associated with the Li site (Fig. 4b). Interestingly, site-specific associations regardless of the different fish species were also visible, for example, *Micrococcaceae* family was significantly associated with the Ce site in both fish species, as well as *Microthrix* genus significantly associated with the Li site in both fish species (Fig. 4a-b).

Clustering analysis based on vst-scaled abundance highlighted the presence of a site-variable gill microbiota, more evident in seabream dataset compared to the seabass dataset, confirming the evidence described in the analyzes above and highlighting the effectiveness of the use of targeted-metagenomic approach to detect site-dependent bacterial signature in different fish species.

## Discussion

The globalization of the seafood trade and the lack of standards for information exchange within the supply chain have made tracking of seafood very challenging [27]. A transparent and traceable seafood supply chain is necessary to promote high-end farmed seafood and to support a *sea-to-fork* scenario. The NGS techniques, in conjunction with other traceability techniques, will better link all the actors involved within the supply chain such as farmers, regulators, policymakers, scientists and consumers. The application of the animal microbiome in the traceability context, for example by using targeted and untargeted metagenomic approaches, will introduce a powerful and replicable tool to pursue designation of origins allowing the usage of guarantee trademark such as Protected Designation of Origin (PDO), Protected Geographical Indication (PGI), or authentication and food fraud detection.

Here, we test the application of a widely used technique, easy to use for a company or institution for the actually restrained cost and execution time, on two very important fish species for their relevance in fish trade in a challenging context represented by fishing areas highly close to each other, performing the targeted metagenomic analysis on gills tissue because they are described to offer a comprehensive signature of the diversity and composition of fish microbiota [25].

Our main findings were resumed by the following observations, (i) The bacterial diversity (alpha and beta diversity) was significantly affected by both fishing species and fishing sites. The fish species was the main driver in shaping the gills microbiota, however specific variations according to different fishing areas were also observed. (ii) Site-dependent changes in bacterial diversity were not equally evident in both fish species, in fact, seabream specimens showed a clear polarization of the gills microbiota according to the three different fishing sites while seabass did not show clear differences between Ce vs. Li sites. This data suggests that the application of these techniques could be affected by the fish species considered, which implies a different fine-tuning of targeted metabarcoding approaches. (iii) The LRT (DESeq2) analysis produced reliable taxonomic profiles which, combined with clustering analysis, provide site-specific clusters based on ASVs abundance distribution.

In detail, all the observations mentioned above were supported by the following specific results. The composition of the gill bacterial communities exhibited a site-dependent variability and it is constrained by a relatively reduced number of core bacterial members associated with each fish species datasets. Our analysis shows that the core is strongly dependent on the fish species under examination: seabream specimen presents a reduced core in terms of ASVs (this condition was driven by high site-specific diversity), while the seabass specimen showed a broader core microbiota (a greater number of ASVs shared by all samples in the sea bass dataset). The greater number of core memberships in the seabass specimens explained the reduced values of variance explained in the beta diversity metrics observed when different fishing sites were compared and the absence of significant difference in the comparison of Ce vs. Li sites. The genus *Psychrobacter* represented one of the most shared taxa within both fish datasets. The osmotolerant *Psychrobacter* spp., identified as a core genus in the seabream in our study, was also found by Quero and collaborators (2023) and assigned to the core members in sea-farmed seabream in the Mediterranean area [23].In Piredda and collaborators (2023), Psychrobacter was described as one of the most representative taxa of Scomber scombrus from FAO areas 27.4, 27.7 and 27.8. Furthermore, *Psychrobacter* was identified as SSOs in the evaluation of tuna spoilage, highlighting its possible role as a spoilage marker [28]. Taxa such as Carnobacterium and Pseudomonas, identified in our dataset can be considered as SSOs for the assessment of fish freshness, moreover abundances of *Escherichia-Shigella* and *Staphylococcus* can be monitored to ensure the fish healthiness. These aspects highlight the plasticity of this sequencing approach in different contexts, all related to fish traceability.

Although a common taxonomic signature is expected in each fish species microbiota, it was also interesting to note the high number of taxa exclusively associated with each fishing site, especially for the seabream dataset (e.g. *Ahrensia*, *Alkalibacterium*, *Alcanivorax*, *Cycloclasticus*, *Cnexibacter* or *Glutamicibacter*). These specific taxa could represent the component of the gill microbiota to consider for future metabarcoding-based analyses, developing an even more predictive fish traceability system. Interestingly, a small number of significantly selected taxa (see LRT) were identified in the same fishing site in both fish species, indicating that the gill microbiota may reflect the microbial pattern of the geographical area of origin. Similar evidence was recently observed also in the mussels *Mytilus galloprovincialis*, which showed site-specific taxonomic patterns [14, 26], corroborating the hypothesis of its possible application as a tool for traceability.

In the future, the number of taxonomic markers could be increased by extending the cohort of fished specimens and expanding the mapped area. By doing that, specific databases could be created for each area in accordance to specific fish species. This idea was suggested by Liu 2020 [29] in the study of soft-shell clams microbiota, highlighting how the research for solutions in traceability context based on the study of microbial communities gathers growing interest in different seafood branches of study. Finally, this study provides new data on the microbiota of the gills of seabass and seabream in a coastal area of interest for the local fish supply chain. This work further highlights the importance of using targeted metagenomic approaches for tracking fish species within the fish supply chain, preventing fraud, errors and ensuring the correct trade and safety consumption. This work demonstrates the possibility to use gill microbiome to differentiate the origin of fish capture at a small geographical scale. The proposal of biomarkers analysis based on the gill microbiota can be integrated within the existing methods for control of the fish supply chain or regulatory agencies guidelines in accordance with the FAO tracking system and the Council Regulation (EC) No. 1379/2013. Furthermore, the results presented in this study can play a fundamental role in promoting the use of these methods to increase the fishing ecological sustainability or in defining safe storage methods, adding value and encouraging the broader concept of the consumption of local fish products.

## Conclusion

This study provides an insight of microbial communities of gill associated wild-caught specimens of two different important commercial species (*D. labrax* and *S. aurata*), demonstrating the possibility to discriminate between the fish species and the sampling site by analyzing the respective microbial signatures. In addition, gills can be easily collected from fresh fish right after fishing and the procedure does not alter the appearance and organoleptic qualities of the product. We therefore suggest that our protocol could be implemented by seafood market stakeholders, such as regulatory agencies or companies. As already suggested by del Rio-Lavin [26] and Liu [29], we also would like to emphasize the necessity of an updated database of seafood-associated microbiota, to improve the possibility of implementing this method and subsequent technical performance and yield by research institutions and agencies.

## Methods

### Fish collecting and gills sampling

Fish specimens were caught by a professional fishing company (San Leopoldo Piccola soc. coop. a r.l Largo Monterosa, 42–58,100 Grosseto) in three different sampling sites within one nautical mile (about 1.8 km) from the coast, in the immediate proximity of three ports located at the following coordinates: Livorno, 43.553634889212105, 10.301929423228817; Cecina, 43.303228810603976, 10.486780667967448; Castiglione della Pescaia, 42.76334583850374, 10.883166525614593 (Fig. 1) belonging to the area of Tuscany coast named “Costadegli Etruschi”, belonging to FAO fishing area 37.1.3, of GFCM Geographical Subarea 9, FAO (2022) [30]. The seabass specimens were collected during late autumn/winter 2022–2023 whereas seabreamwas collected during late summer/autumn 2022 in accordance with the seasonality of each fish species. Frozen samples provided by the fishing company were delivered to the Department of Biology of the University of Florence (Italy) and stored at -20 °C until subsequent downstream procedures. After completely thawing the fish, a biopsy of the right gills of about 2 cm^2^ was performed, including both cartilage and lamellar tissue according to methodological procedures mentioned in Clinton 2021 [25]. For each sampling site, ten fish specimens of each fish species were processed, for a total of 60 different individuals. All sampling procedures were carried out in sterile conditions under biological hood and all instruments have been previously sterilized with ethanol 70% to avoid any contamination [31].

### DNA extraction, library preparation and targeted metagenomic sequencing

Genomic DNA was extracted from gills tissue by DNeasy Powersoil Kit (Qiagen, Hiledn, Germany), according to manufacturer instructions. The genomic DNA was visualized for quality and integrity on stained agarose gel and quantified using Qubit 4 Fluorometer (Thermo Fisher Scientific,Waltham, MA USA) 1x dsDNA High Sensitivity kit. For each sample, PCR amplification of 16 S V3-V4 hypervariable regions was performed, using primers 341f (5′-CCTACGGGNGGCWGCAG‐3)′ and 805r (5′‐GACTACNVGGGTWTCTAATCC‐3′) [32]. Libraries were prepared according to Illumina Protocol *16 S Metagenomic Sequencing Library Preparation* (Part # 15,044,223 Rev. B; URL: https://www.illumina.com/content/dam/illumina-support/documents/documentation/chemistry_documentation/16s/16s-metagenomic-library-prep-guide-15044223-b.pdf). Paired end 2 × 300 bp were performed on Illumina MiSeq Platform (Illumina Inc) using MiSeq Reagent Kit v3 (600 cycle).

### Amplicon sequence variants production and statistical analysis

The primer pair sequences were removed by using cutadapt version 3.5 [33]. The amplicon sequence variants (ASVs) inference was assessed by DADA2 pipeline version 1.26 [34]. Low quality sequences were removed by using “filterAndTrim” with a maximum number of expected error thresholds of 2 for forward and reverse read pairs. The error rate estimation was performed using the “learnErrors” and denoising was assessed by the “dada” function with default parameters. Denoised sequences were merged by using the “mergePairs” function and chimeric sequences were removed using the “removeBimeraDenovo” function. The taxonomic classification was inferred by using DECIPHER package version 2.26 [35]. ASVs that were not assigned to bacteria (unknown) or assigned to chloroplasts and mitochondria sequences were removed to properly perform the downstream statistical analyses.

Statistical analyzes were performed in the R environment version 4.2.2 [36]. Beta diversity was explored by using the “vegan” package version 2.6.4 [37]. Rarefaction curves were calculated and depicted by using the “ggrare” function of “ranacapa” package version 0.1 [38]. Core microbiome was assessed by using the “microbiome” package version 1.20 [39]. After removal of single counts and transformation into relative abundances, the distribution of samples was visualized by Principal Coordinate Analysis (PCoA) using the “cmdscale” function of the “stat” package based on Bray-Curtis distance index. Permutational multivariate analysis of variance using distance matrices (adonis permanova) was performed to inspect differences between sample groups using the “adonis2” function of the “vegan” package version 2.6.4. Pairwise comparisons on variance among different sites were assessed using pairwise adonis permanova by using the “pairwise.adonis” function from “pairwiseAdonis” package version 0.4 [40]. Alpha diversity measures were produced by the “estimate_richness” function from the “phyloseq” package version 1.42 [41] while Evennes was defined as the index/log of Shannon diversity (observed richness). Significant differences among groups were assessed by using the Wilcoxon pairwise test using the “wilcox_test” function from the “rstatix” package version 0.7.2 [42]. Abundance-occupancy analysis was performed to detect core membership among fishing sites [43]. Core microbiota were assessed on compositional-transformed abundances by using the “plot_core” function of “microbiome” package version 1.20 [39].

Differential abundance analysis was performed using the likelihood ratio test (LRT) implemented in the DESeq2 package version 1.38.3 [44]. Before the LRT test, singletons were removed to mitigate the hypothesis that extremely rare species could be considered the main drivers of differences between the groups. The size factor estimate was calculated using the “postcount” method to estimate geometric means of ASVs in the presence of zeros using the “estimateSizeFactors” function of the “DESeq2” package. ASVs abundance values depicted in the ternary space were calculated using the “microbiomeutilities” package version 1.0.17 [45] with default parameters (abundant threshold = 0.0001, prevalence threshold = 0.1) then displayed by using “ggtern” package version 3.4.2 [46]. The figures were produced using the “ggplot2” package version 3.4.2 [47] and edited using the open source graphics editor Inkscape 1.1.2 (http://inkscape.org/).

### Electronic supplementary material

Below is the link to the electronic supplementary material.

Supplementary Material 1

Supplementary Material 2

Supplementary Material 3

Supplementary Material 4

Supplementary Material 5

Supplementary Material 6

Supplementary Material 7

Supplementary Material 8

## Data Availability

The 16 S rRNA raw sequences have been deposited to the European Nucleotide Archive (ENA) under the accession code PRJEB67338. All results from statistical analyses were mentioned by writing in the main text, reported as figures or tables in the main text and supplementary materials. Any other additional information can be provided upon request to the corresponding author.
