# Supplementary material for: Enhancing seafood traceability: tracking the origin of seabass and seabream from the tuscan coast area by the analysis of the gill bacterial communities
Source: Anim Microbiome. 2024 Mar 14;6:13. doi: 10.1186/s42523-024-00300-z (PMC10938666; doi:10.1186/s42523-024-00300-z)
Supplement: Supplementary file 1 — Supplementary Material 1 [file 42523_2024_300_MOESM1_ESM.docx]

**Supplementary materials**

**Enhancing seafood traceability: tracking the origin of seabass and seabream from the Tuscan coast area by the analysis of the gill bacterial communities**

Niccolò Meriggi^1^**†,** Alessandro Russo^1^**†**, Sonia Renzi^1^, Benedetta Cerasuolo^1^, Marta Nerini^1^, Alberto Ugolini^2^, Massimiliano Marvasi^1^, Duccio Cavalieri^1^*

**†** authors who equally contributed to this work.

* Corresponding author.

^1^ Department of Biology, University of Florence, Sesto Fiorentino, IT50019, Italy.

^2^ Department of Biology, University of Florence, Florence, IT50125, Italy.

**Table S1.** Biometric data of the caught samples. Table reports mean and standard deviation (s.d.) of biometric data, i.e. weight and length, of the fish samples grouped according to each fishing site for the two fish species. The fishing sites are reported according to the following legend, CdP: Castiglione della Pescaia, Ce: Cecina, Li: Livorno.

| **Fish species** | **Sampling site** |  | **Weight (g)** | **Lenght (cm)** |
| --- | --- | --- | --- | --- |
| Seabream | CdP | *mean* | 577 | 31.53 |
|  |  | *s.d.* | 127.67 | 2.88 |
|  | Li | *mean* | 274.7 | 24.77 |
|  |  | *s.d.* | 28.55 | 0.88 |
|  | Ce | *mean* | 212.8 | 25.05 |
|  |  | *s.d.* | 21.05 | 1.12 |
| Seabass | CdP | *mean* | 330.4 | 32.25 |
|  |  | *s.d.* | 93.88 | 2.98 |
|  | Li | mean | 402.1 | 30.85 |
|  |  | *s.d.* | 64.92 | 2,39 |
|  | Ce | *mean* | 233.6 | 28.64 |
|  |  | *s.d.* | 47.2 | 1.87 |


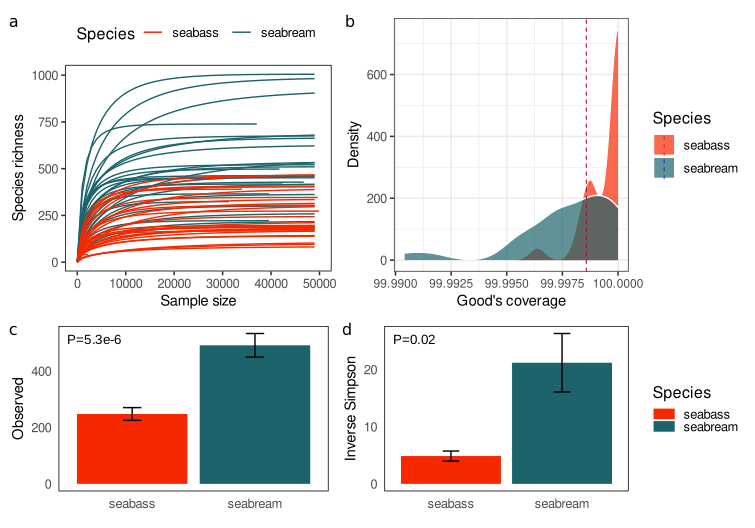


**Figure S1.** Bacterial diversity among fish species. **a** Rarefaction curves report the total number of ASVs (Species richness) and total number of reads (Sample size) according to the different fish species. **b** Distribution of good’s coverage values (Density) among different fish species. The x-axis reports the good’s coverage values and the dashed line reports the mean values for each fish species. **c**, **d** Barplots represent the alpha diversity metrics represented by total number of ASVs (**c**) and Inverse Simpson index (**d**) compared among the two fish species. P-value after Kruskal-wallis test is reported inside the panels. Standard errors are reported by using errorbars.

**Table S2.** Pairwise adonis permanova results. The table reports F-model and variance explained (R^2^) calculated for each site group pair according to each fish species. Significant pairs are reported using asterisks (*; P<0.05, **; P<0.01). The fishing sites are reported according to the following legend, CdP: Castiglione della Pescaia, Ce: Cecina, Li: Livorno.

| ***Sparus aurata*** | | |
| --- | --- | --- |
| Pair | F-Model | R^2^ |
| Ce vs CdP | 2.5371 | 0.1235 * |
| Ce vs Li | 13.0416 | 0.4201 ** |
| CdP vs Li | 16.3734 | 0.4763 ** |
| ***Dicentrarchus labrax*** | | |
| Pair | F-Model | R^2^ |
| Ce vs CdP | 8.0783 | 0.3355 ** |
| Ce vs Li | 0.8167 | 0.0434 |
| CdP vs Li | 11.1283 | 0.4102 ** |


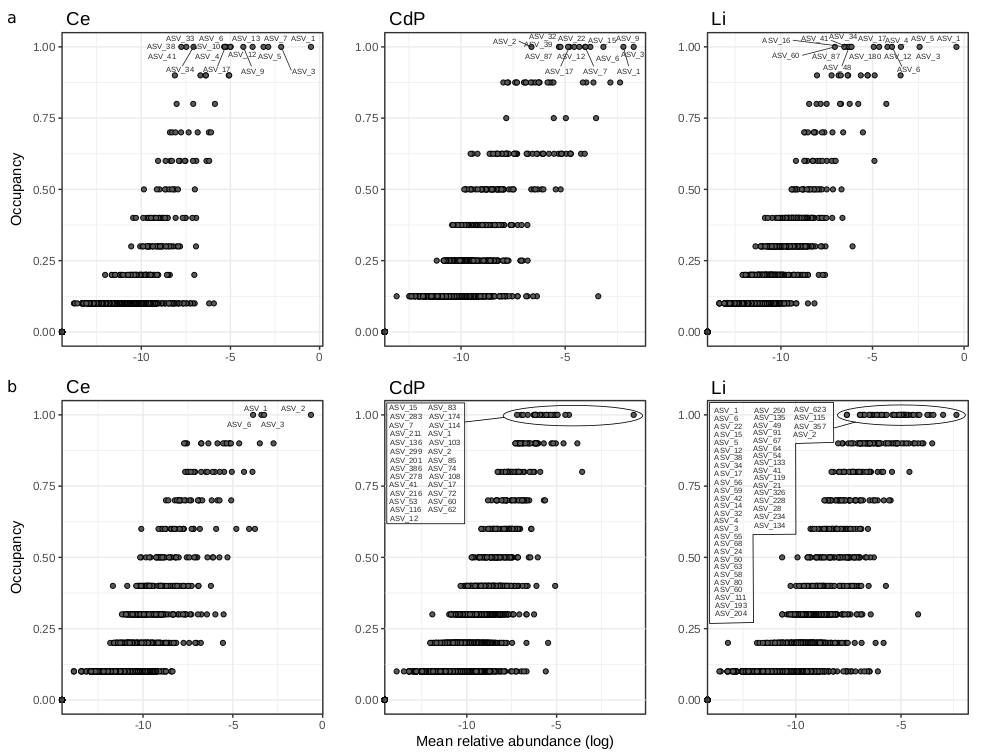


**Figure S2.** Abundance-occupancy ratio. Abundance-occupancy ratio shows the core bacterial members of the fishing sites for the seabass (**a**) and seabream (**b**) datasets. The dots represent the ASVs and the core members (Occupancy =1) are highlighted inside each panel. The occupancy values are reported on the y-axis while the logarithm of the mean relative abundances are reported on the x-axis. The fishing sites are reported according to the following legend, CdP: Castiglione della Pescaia, Ce: Cecina, Li: Livorno.

**Additional file 1: Table S3.** Top core members selected by abundance-occupancy analysis. Abundance-occupancy table reports the top core bacterial members (Occupancy =1) obtained from occupancy-abundance analysis and highlighted in figure S2. Each core member (ASV) is reported with the mean relative abundance values and related taxonomic assignment at the deepest taxonomic level for each site and fish species. The fishing sites are reported according to the following legend, CdP: Castiglione della Pescaia, Ce: Cecina, Li: Livorno.


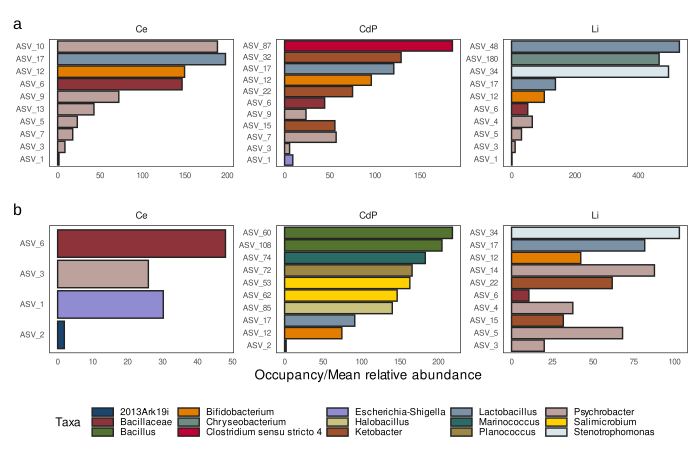


**Figure S3.** Top members with the highest contribution to the occupancy-abundance ratio. Barplots report the occupancy-mean abundance ratio (occupancy/mean relative abundance) rate of top ten ASVs selected from occupancy-abundance analysis associated with each fishing site and performed in seabass (**a**) and seabream (**b**) datasets. ASV -related taxonomic assignment is reported according to the color scheme in the legend. The fishing sites are reported according to the following legend, CdP: Castiglione della Pescaia, Ce: Cecina, Li: Livorno.

**Additional file 2: Table S4.** Results from sequence variant clustering (LRT of DESeq2). Table reported the results from LRT analysis performed in seabass and seabream datasets. Columns reported the name of ASV tested (asv), the mean overall abundance (baseMean), the logarithm of the ratio between two levels of the factor considered, the standard error of the log2FoldChange (lfcSE), the difference in deviance between the reduced model and the full model (stat), p-value obtained by comparing stat to a chi-squared distribution (pvalue) and p-value after Benjamini–Hochberg adjustment (padj). ASV taxonomic assignment from Domain to Species is also reported.


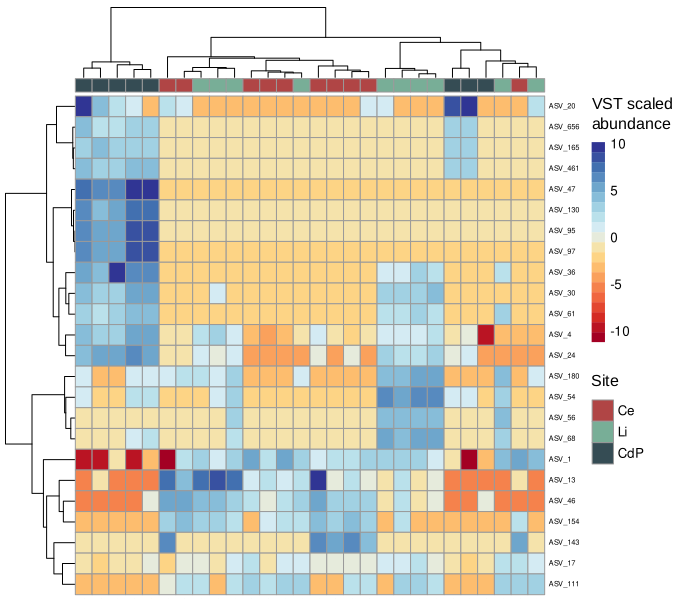


**Figure S4.** Sequence variants clustering across different fishing sites in seabass dataset. ASVs that significantly vary within fishing sites (LRT of DESeq2) are clustered according to their variance-stabilized abundance (VST scaled abundance). ASVs clustering is produced according to Ward D2 method and rows/columns branches are built using euclidean distance. ASVs scaled abundance values are reported using color gradients reported in the legend. ASVs are reported on the heatmap rows while samples are reported on the columns. The fishing sites are reported according to the following legend, CdP: Castiglione della Pescaia, Ce: Cecina, Li: Livorno.

**Additional file 3: Figure S5.** Sequence variants clustering across different fishing sites in seabream dataset. ASVs that significantly vary within fishing sites (LRT of DESeq2) are clustered according to their variance-stabilized abundance (VST scaled abundance). ASVs clustering is produced according to Ward D2 method and rows/columns branches are built using euclidean distance. ASVs scaled abundance values are reported using color gradients reported in the legend. ASVs are reported on the heatmap rows while samples are reported on the columns. The fishing sites are reported according to the following legend, CdP: Castiglione della Pescaia, Ce: Cecina, Li: Livorno.
