# Supplementary figures and images for: Enhancing seafood traceability: tracking the origin of seabass and seabream from the tuscan coast area by the analysis of the gill bacterial communities
Source: Anim Microbiome. 2024 Mar 14;6:13. doi: 10.1186/s42523-024-00300-z (PMC10938666; doi:10.1186/s42523-024-00300-z)

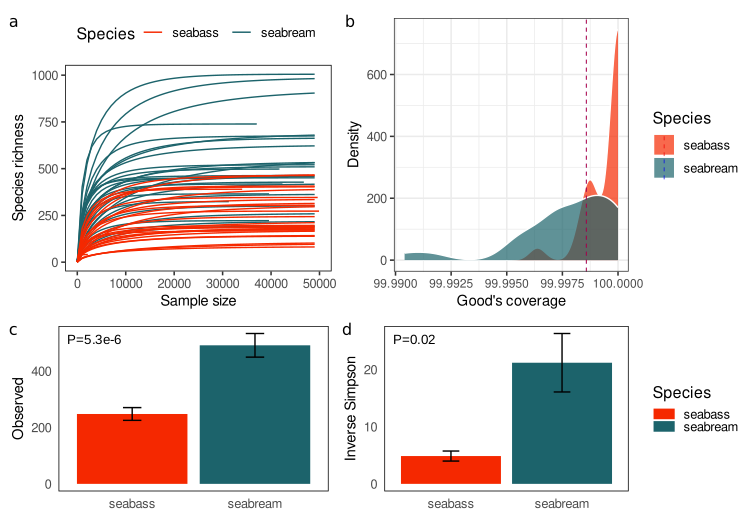

Supplement: Supplementary file 2 — Supplementary Material 2 [file 42523_2024_300_MOESM2_ESM.png]

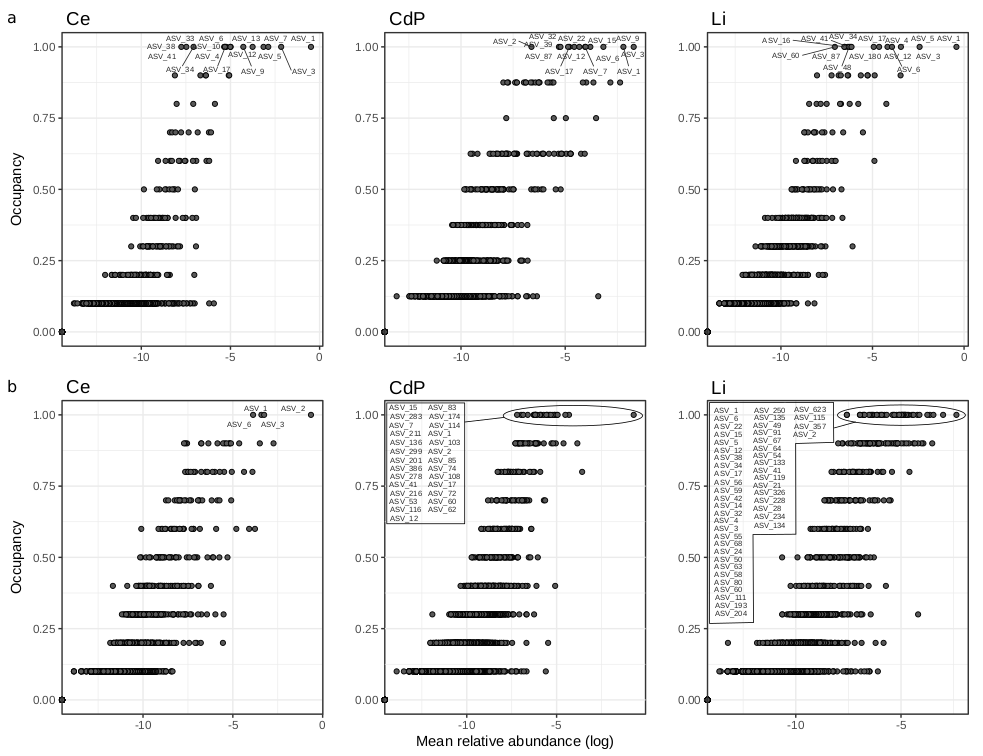

Supplement: Supplementary file 3 — Supplementary Material 3 [file 42523_2024_300_MOESM3_ESM.png]

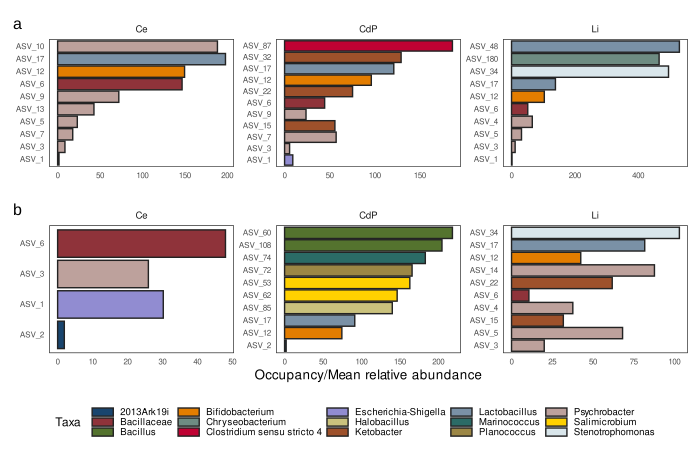

Supplement: Supplementary file 4 — Supplementary Material 4 [file 42523_2024_300_MOESM4_ESM.png]

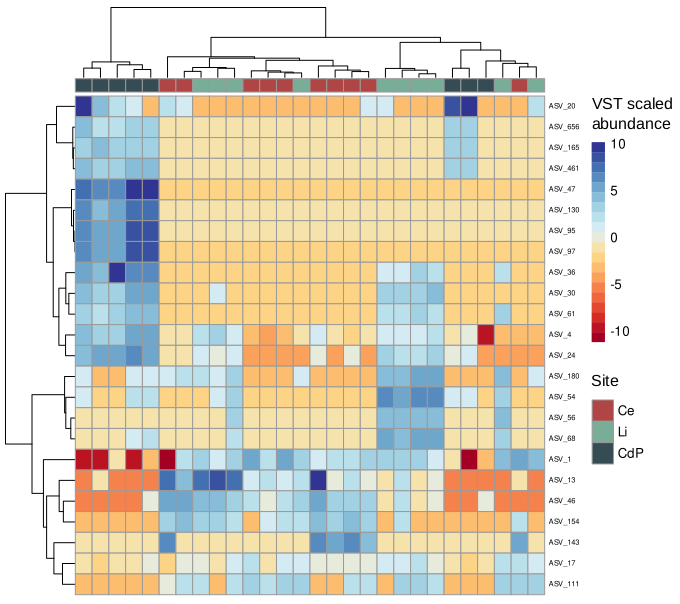

Supplement: Supplementary file 5 — Supplementary Material 5 [file 42523_2024_300_MOESM5_ESM.png]
